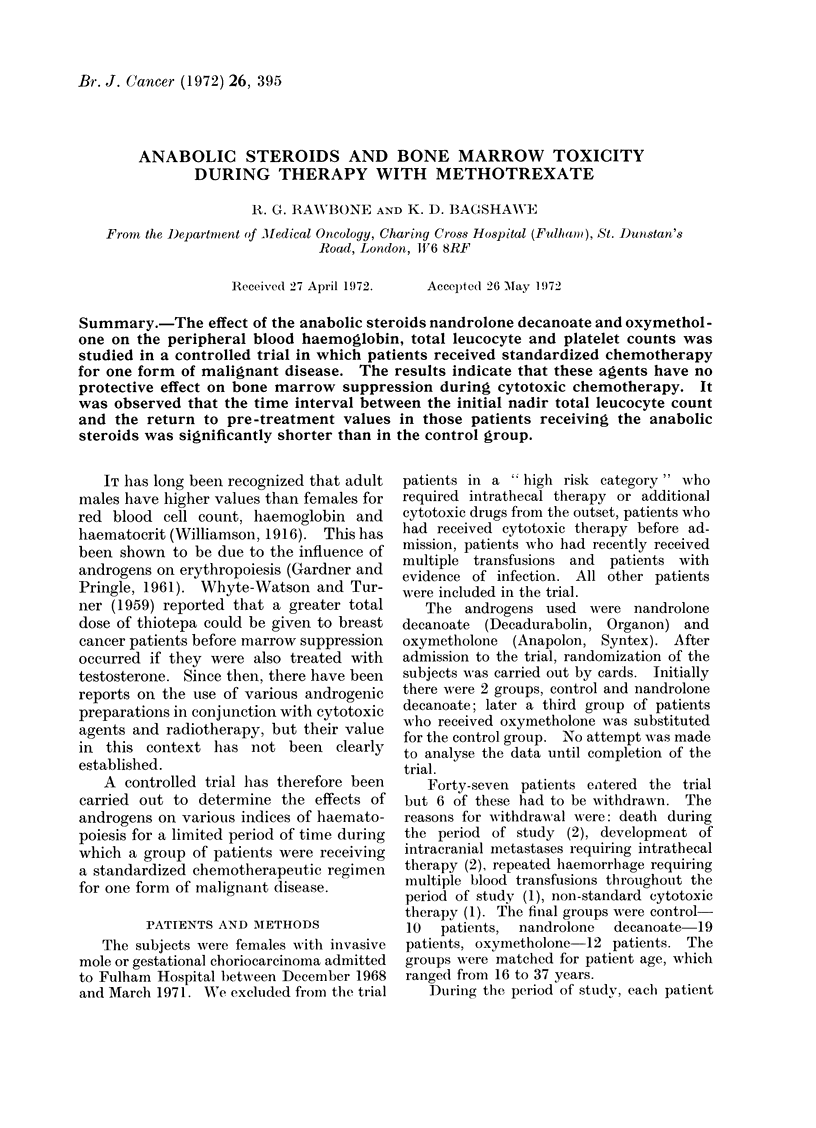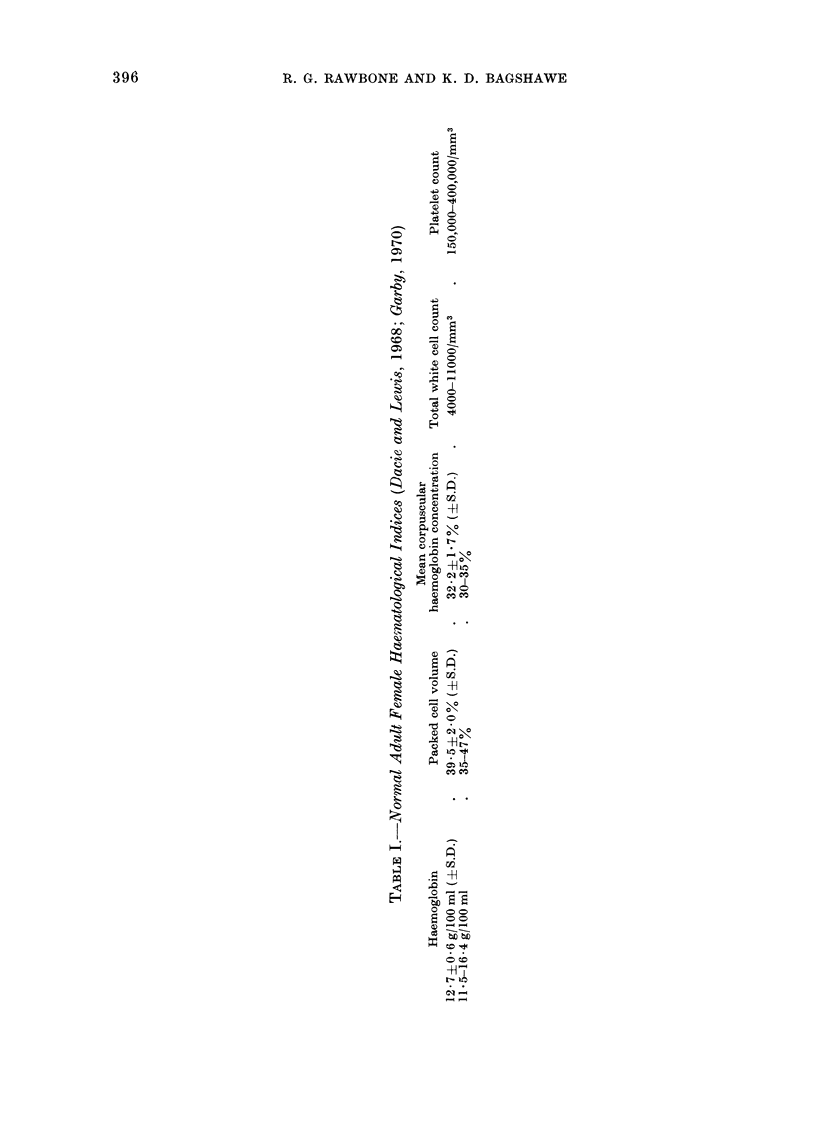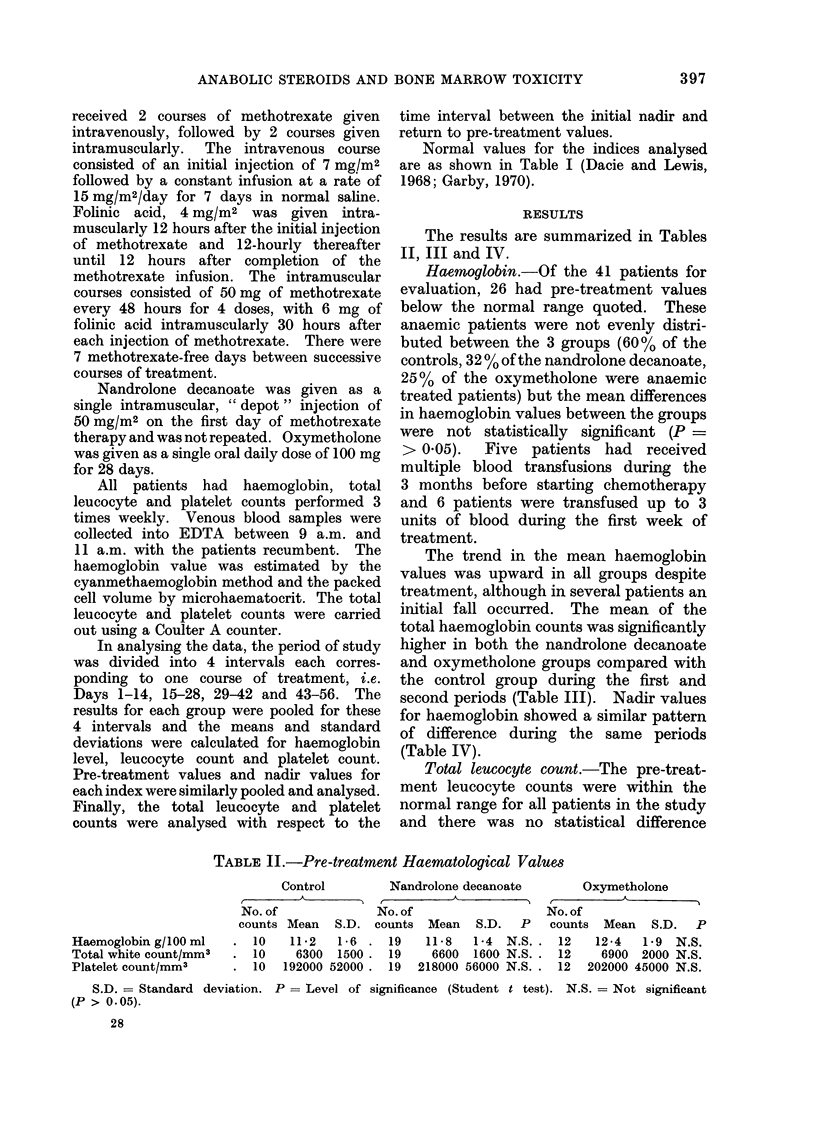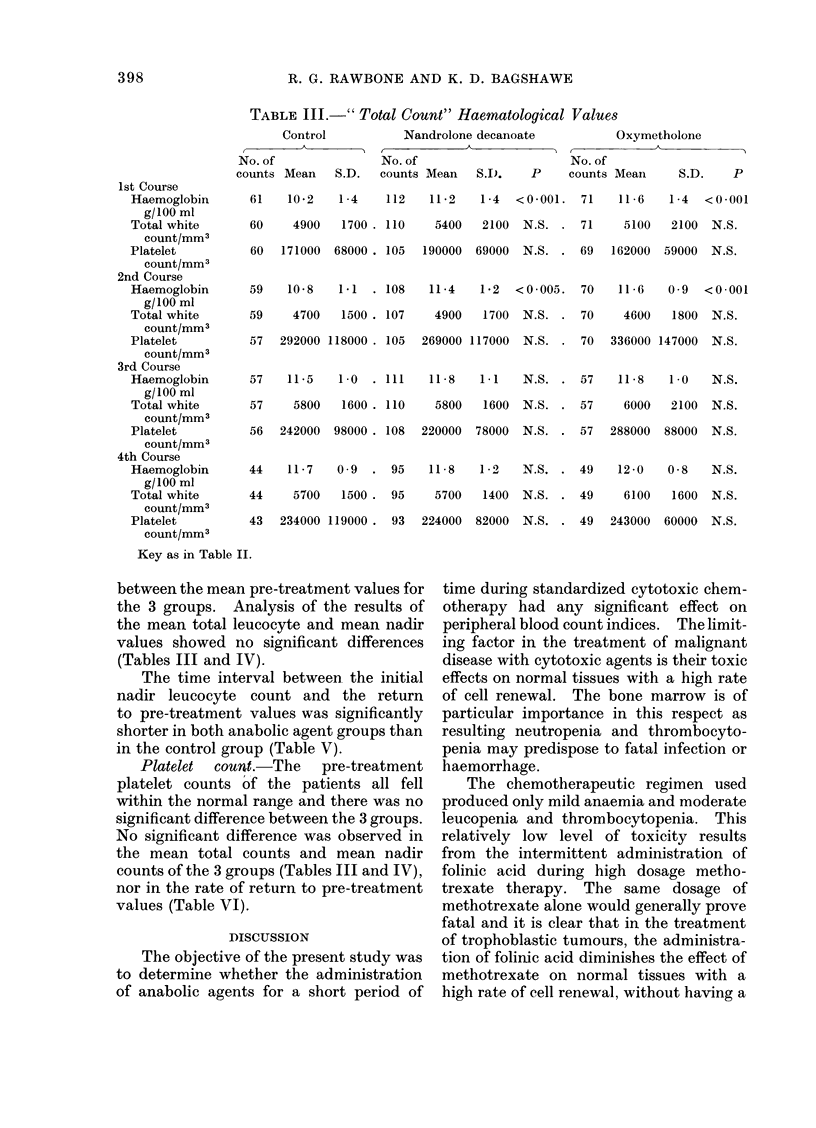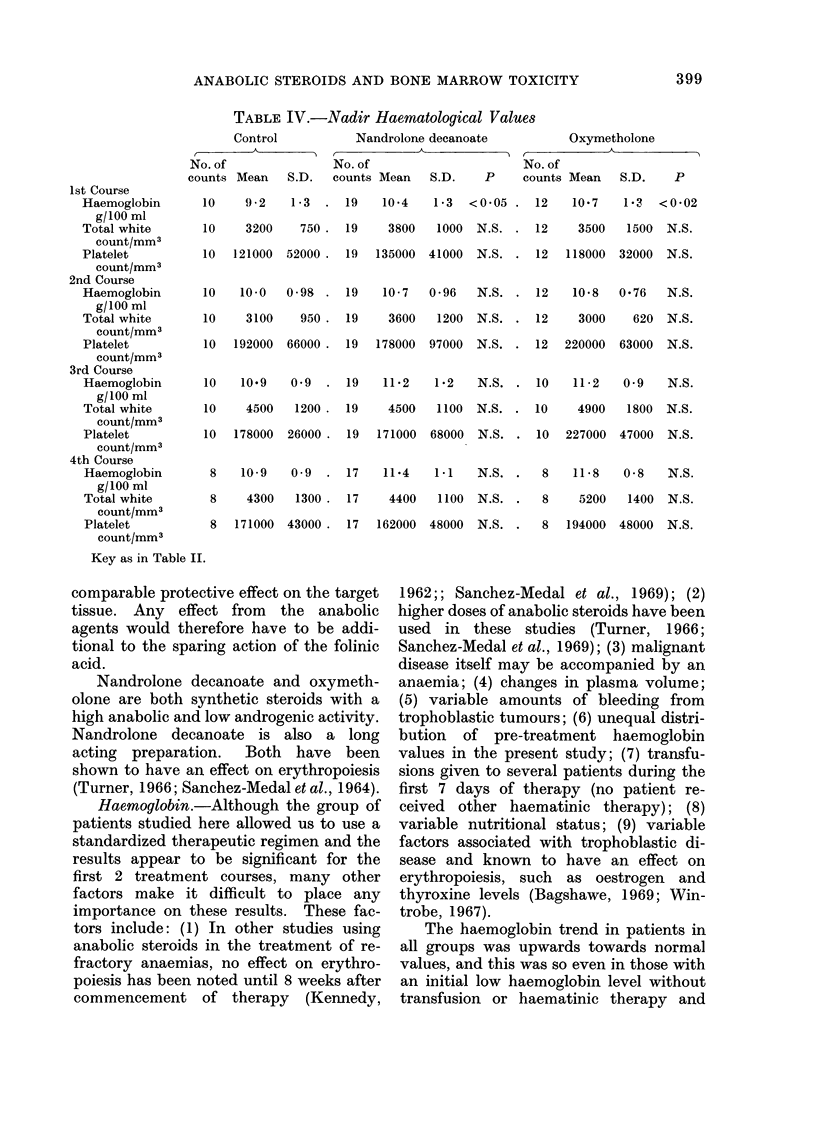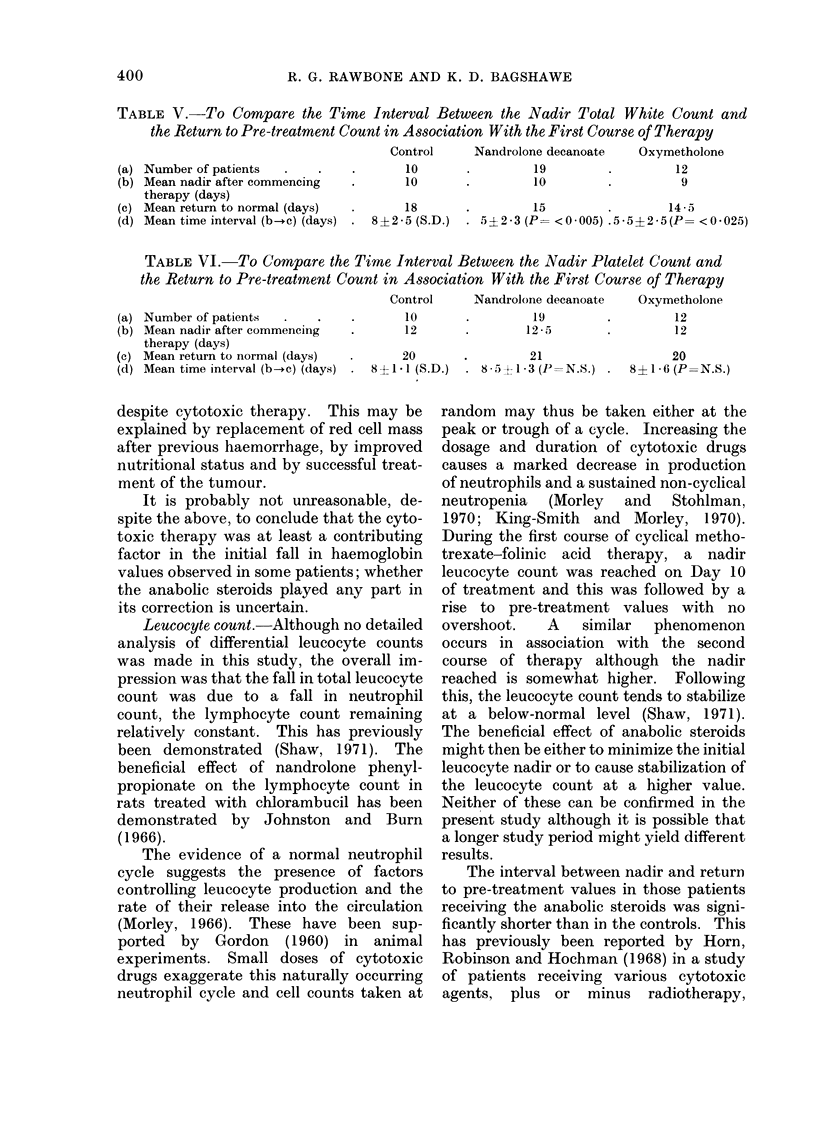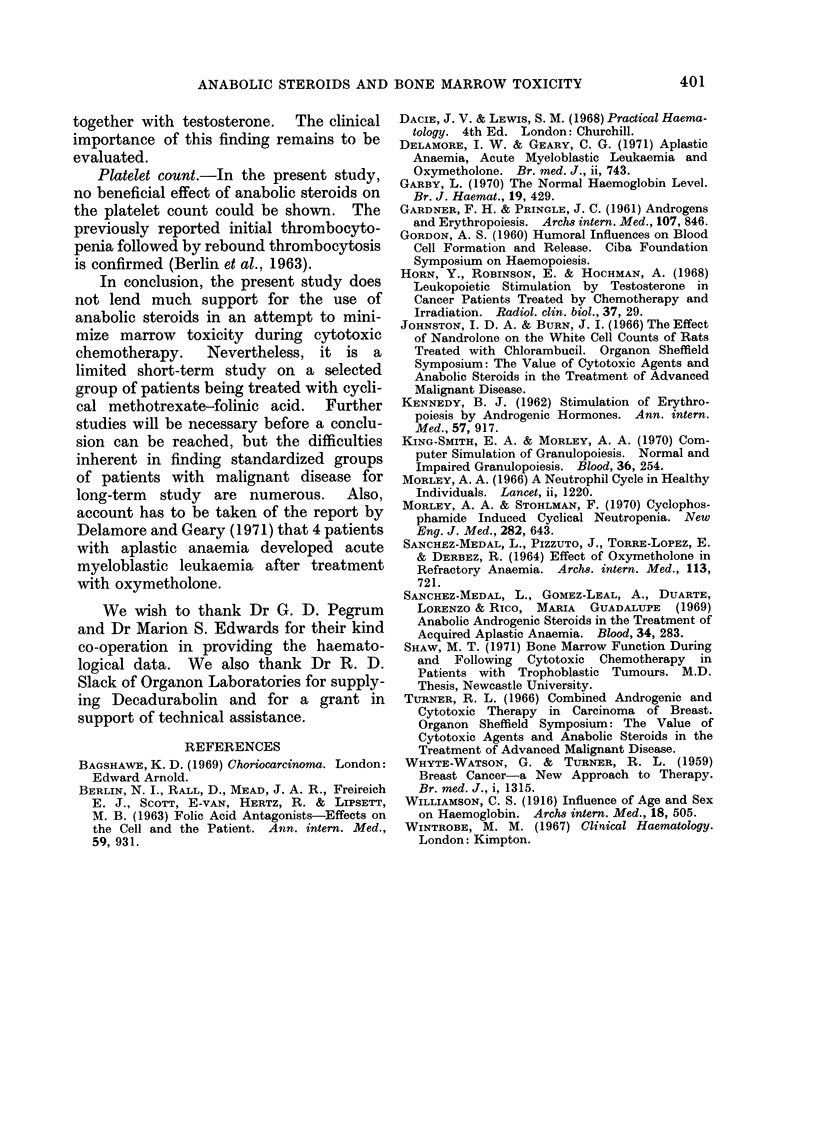# Anabolic Steroids and Bone Marrow Toxicity During Therapy with Methotrexate

**DOI:** 10.1038/bjc.1972.52

**Published:** 1972-10

**Authors:** R. G. Rawbone, K. D. Bagshawe

## Abstract

The effect of the anabolic steroids nandrolone decanoate and oxymetholone on the peripheral blood haemoglobin, total leucocyte and platelet counts was studied in a controlled trial in which patients received standardized chemotherapy for one form of malignant disease. The results indicate that these agents have no protective effect on bone marrow suppression during cytotoxic chemotherapy. It was observed that the time interval between the initial nadir total leucocyte count and the return to pre-treatment values in those patients receiving the anabolic steroids was significantly shorter than in the control group.


*Br. J. Cancer* (1972) **26**, 395

### ANABOLIC STEROIDS AND BONE MARROW TOXICITY DURING THERAPY WITH METHOTREXATE

R. G. RAWBONE AND K. D. BAGSHAWE

*From the Department of Medical Oncology, Charing Cross Hospital (Fulham), St. Dunstan's Road, London, W6 8RF*

Received 27 April 1972. Accepted 26 May 1972

**Summary.—The effect of the anabolic steroids nandrolone decanoate and oxymethol-one on the peripheral blood haemoglobin, total leucocyte and platelet counts was studied in a controlled trial in which patients received standardized chemotherapy for one form of malignant disease. The results indicate that these agents have no protective effect on bone marrow suppression during cytotoxic chemotherapy. It was observed that the time interval between the initial nadir total leucocyte count and the return to pre-treatment values in those patients receiving the anabolic steroids was significantly shorter than in the control group.**

IT has long been recognized that adult males have higher values than females for red blood cell count, haemoglobin and haematocrit (Williamson, 1916). This has been shown to be due to the influence of androgens on erythropoiesis (Gardner and Pringle, 1961). Whyte-Watson and Turner (1959) reported that a greater total dose of thiotepa could be given to breast cancer patients before marrow suppression occurred if they were also treated with testosterone. Since then, there have been reports on the use of various androgenic preparations in conjunction with cytotoxic agents and radiotherapy, but their value in this context has not been clearly established.

A controlled trial has therefore been carried out to determine the effects of androgens on various indices of haematopoiesis for a limited period of time during which a group of patients were receiving a standardized chemotherapeutic regimen for one form of malignant disease.

### PATIENTS AND METHODS

The subjects were females with invasive mole or gestational choriocarcinoma admitted to Fulham Hospital between December 1968 and March 1971. We excluded from the trial patients in a "high risk category" who required intrathecal therapy or additional cytotoxic drugs from the outset, patients who had received cytotoxic therapy before admission, patients who had recently received multiple transfusions and patients with evidence of infection. All other patients were included in the trial.

The androgens used were nandrolone decanoate (Decadurabolin, Organon) and oxymetholone (Anapolon, Syntex). After admission to the trial, randomization of the subjects was carried out by cards. Initially there were 2 groups, control and nandrolone decanoate; later a third group of patients who received oxymetholone was substituted for the control group. No attempt was made to analyse the data until completion of the trial.

Forty-seven patients entered the trial but 6 of these had to be withdrawn. The reasons for withdrawal were: death during the period of study (2), development of intracranial metastases requiring intrathecal therapy (2), repeated haemorrhage requiring multiple blood transfusions throughout the period of study (1), non-standard cytotoxic therapy (1). The final groups were control—10 patients, nandrolone decanoate—19 patients, oxymetholone—12 patients. The groups were matched for patient age, which ranged from 16 to 37 years.

During the period of study, each patient

TABLE I.—*Normal Adult Female Haematological Indices* (*Dacie and Lewis*, 1968; *Garby*, 1970)

| Haemoglobin | Packed cell volume | Mean corpuscular haemoglobin concentration | Total white cell count | Platelet count |
|---|---|---|---|---|
| 12·7±0·6 g/100 ml (±S.D.) | 39·5±2·0% (±S.D.) | 32·2±1·7% (±S.D.) | 4000–11000/mm³ | 150,000–400,000/mm³ |
| 11·5–16·4 g/100 ml | 35–47% | 30–35% | | |

received 2 courses of methotrexate given intravenously, followed by 2 courses given intramuscularly. The intravenous course consisted of an initial injection of 7 mg/m$^2$ followed by a constant infusion at a rate of 15 mg/m$^2$/day for 7 days in normal saline. Folinic acid, 4 mg/m$^2$ was given intramuscularly 12 hours after the initial injection of methotrexate and 12-hourly thereafter until 12 hours after completion of the methotrexate infusion. The intramuscular courses consisted of 50 mg of methotrexate every 48 hours for 4 doses, with 6 mg of folinic acid intramuscularly 30 hours after each injection of methotrexate. There were 7 methotrexate-free days between successive courses of treatment.

Nandrolone decanoate was given as a single intramuscular, "depot" injection of 50 mg/m$^2$ on the first day of methotrexate therapy and was not repeated. Oxymetholone was given as a single oral daily dose of 100 mg for 28 days.

All patients had haemoglobin, total leucocyte and platelet counts performed 3 times weekly. Venous blood samples were collected into EDTA between 9 a.m. and 11 a.m. with the patients recumbent. The haemoglobin value was estimated by the cyanmethaemoglobin method and the packed cell volume by microhaematocrit. The total leucocyte and platelet counts were carried out using a Coulter A counter.

In analysing the data, the period of study was divided into 4 intervals each corresponding to one course of treatment, *i.e.* Days 1–14, 15–28, 29–42 and 43–56. The results for each group were pooled for these 4 intervals and the means and standard deviations were calculated for haemoglobin level, leucocyte count and platelet count. Pre-treatment values and nadir values for each index were similarly pooled and analysed. Finally, the total leucocyte and platelet counts were analysed with respect to the time interval between the initial nadir and return to pre-treatment values.

Normal values for the indices analysed are as shown in Table I (Dacie and Lewis, 1968; Garby, 1970).

### RESULTS

The results are summarized in Tables II, III and IV.

*Haemoglobin.*—Of the 41 patients for evaluation, 26 had pre-treatment values below the normal range quoted. These anaemic patients were not evenly distributed between the 3 groups (60% of the controls, 32% of the nandrolone decanoate, 25% of the oxymetholone were anaemic treated patients) but the mean differences in haemoglobin values between the groups were not statistically significant ($P = > 0{\cdot}05$). Five patients had received multiple blood transfusions during the 3 months before starting chemotherapy and 6 patients were transfused up to 3 units of blood during the first week of treatment.

The trend in the mean haemoglobin values was upward in all groups despite treatment, although in several patients an initial fall occurred. The mean of the total haemoglobin counts was significantly higher in both the nandrolone decanoate and oxymetholone groups compared with the control group during the first and second periods (Table III). Nadir values for haemoglobin showed a similar pattern of difference during the same periods (Table IV).

*Total leucocyte count.*—The pre-treatment leucocyte counts were within the normal range for all patients in the study and there was no statistical difference

TABLE II.—*Pre-treatment Haematological Values*

| | Control | | | Nandrolone decanoate | | | | Oxymetholone | | | |
|---|---|---|---|---|---|---|---|---|---|---|---|
| | No. of counts | Mean | S.D. | No. of counts | Mean | S.D. | $P$ | No. of counts | Mean | S.D. | $P$ |
| Haemoglobin g/100 ml | 10 | 11·2 | 1·6 | 19 | 11·8 | 1·4 | N.S. | 12 | 12·4 | 1·9 | N.S. |
| Total white count/mm$^3$ | 10 | 6300 | 1500 | 19 | 6600 | 1600 | N.S. | 12 | 6900 | 2000 | N.S. |
| Platelet count/mm$^3$ | 10 | 192000 | 52000 | 19 | 218000 | 56000 | N.S. | 12 | 202000 | 45000 | N.S. |

S.D. = Standard deviation. $P$ = Level of significance (Student $t$ test). N.S. = Not significant ($P > 0.05$).

TABLE III.—"*Total Count*" *Haematological Values*

| | Control | | | Nandrolone decanoate | | | | Oxymetholone | | | |
|---|---|---|---|---|---|---|---|---|---|---|---|
| | No. of counts | Mean | S.D. | No. of counts | Mean | S.D. | *P* | No. of counts | Mean | S.D. | *P* |
| 1st Course | | | | | | | | | | | |
| Haemoglobin g/100 ml | 61 | 10·2 | 1·4 | 112 | 11·2 | 1·4 | <0·001 | 71 | 11·6 | 1·4 | <0·001 |
| Total white count/mm³ | 60 | 4900 | 1700 | 110 | 5400 | 2100 | N.S. | 71 | 5100 | 2100 | N.S. |
| Platelet count/mm³ | 60 | 171000 | 68000 | 105 | 190000 | 69000 | N.S. | 69 | 162000 | 59000 | N.S. |
| 2nd Course | | | | | | | | | | | |
| Haemoglobin g/100 ml | 59 | 10·8 | 1·1 | 108 | 11·4 | 1·2 | <0·005 | 70 | 11·6 | 0·9 | <0·001 |
| Total white count/mm³ | 59 | 4700 | 1500 | 107 | 4900 | 1700 | N.S. | 70 | 4600 | 1800 | N.S. |
| Platelet count/mm³ | 57 | 292000 | 118000 | 105 | 269000 | 117000 | N.S. | 70 | 336000 | 147000 | N.S. |
| 3rd Course | | | | | | | | | | | |
| Haemoglobin g/100 ml | 57 | 11·5 | 1·0 | 111 | 11·8 | 1·1 | N.S. | 57 | 11·8 | 1·0 | N.S. |
| Total white count/mm³ | 57 | 5800 | 1600 | 110 | 5800 | 1600 | N.S. | 57 | 6000 | 2100 | N.S. |
| Platelet count/mm³ | 56 | 242000 | 98000 | 108 | 220000 | 78000 | N.S. | 57 | 288000 | 88000 | N.S. |
| 4th Course | | | | | | | | | | | |
| Haemoglobin g/100 ml | 44 | 11·7 | 0·9 | 95 | 11·8 | 1·2 | N.S. | 49 | 12·0 | 0·8 | N.S. |
| Total white count/mm³ | 44 | 5700 | 1500 | 95 | 5700 | 1400 | N.S. | 49 | 6100 | 1600 | N.S. |
| Platelet count/mm³ | 43 | 234000 | 119000 | 93 | 224000 | 82000 | N.S. | 49 | 243000 | 60000 | N.S. |

Key as in Table II.

between the mean pre-treatment values for the 3 groups. Analysis of the results of the mean total leucocyte and mean nadir values showed no significant differences (Tables III and IV).

The time interval between the initial nadir leucocyte count and the return to pre-treatment values was significantly shorter in both anabolic agent groups than in the control group (Table V).

*Platelet count*.—The pre-treatment platelet counts of the patients all fell within the normal range and there was no significant difference between the 3 groups. No significant difference was observed in the mean total counts and mean nadir counts of the 3 groups (Tables III and IV), nor in the rate of return to pre-treatment values (Table VI).

### DISCUSSION

The objective of the present study was to determine whether the administration of anabolic agents for a short period of time during standardized cytotoxic chemotherapy had any significant effect on peripheral blood count indices. The limiting factor in the treatment of malignant disease with cytotoxic agents is their toxic effects on normal tissues with a high rate of cell renewal. The bone marrow is of particular importance in this respect as resulting neutropenia and thrombocytopenia may predispose to fatal infection or haemorrhage.

The chemotherapeutic regimen used produced only mild anaemia and moderate leucopenia and thrombocytopenia. This relatively low level of toxicity results from the intermittent administration of folinic acid during high dosage methotrexate therapy. The same dosage of methotrexate alone would generally prove fatal and it is clear that in the treatment of trophoblastic tumours, the administration of folinic acid diminishes the effect of methotrexate on normal tissues with a high rate of cell renewal, without having a

TABLE IV.—*Nadir Haematological Values*

| | Control | | | Nandrolone decanoate | | | | Oxymetholone | | | |
|---|---|---|---|---|---|---|---|---|---|---|---|
| | No. of counts | Mean | S.D. | No. of counts | Mean | S.D. | *P* | No. of counts | Mean | S.D. | *P* |
| 1st Course | | | | | | | | | | | |
| Haemoglobin g/100 ml | 10 | 9·2 | 1·3 | 19 | 10·4 | 1·3 | <0·05 | 12 | 10·7 | 1·3 | <0·02 |
| Total white count/mm³ | 10 | 3200 | 750 | 19 | 3800 | 1000 | N.S. | 12 | 3500 | 1500 | N.S. |
| Platelet count/mm³ | 10 | 121000 | 52000 | 19 | 135000 | 41000 | N.S. | 12 | 118000 | 32000 | N.S. |
| 2nd Course | | | | | | | | | | | |
| Haemoglobin g/100 ml | 10 | 10·0 | 0·98 | 19 | 10·7 | 0·96 | N.S. | 12 | 10·8 | 0·76 | N.S. |
| Total white count/mm³ | 10 | 3100 | 950 | 19 | 3600 | 1200 | N.S. | 12 | 3000 | 620 | N.S. |
| Platelet count/mm³ | 10 | 192000 | 66000 | 19 | 178000 | 97000 | N.S. | 12 | 220000 | 63000 | N.S. |
| 3rd Course | | | | | | | | | | | |
| Haemoglobin g/100 ml | 10 | 10·9 | 0·9 | 19 | 11·2 | 1·2 | N.S. | 10 | 11·2 | 0·9 | N.S. |
| Total white count/mm³ | 10 | 4500 | 1200 | 19 | 4500 | 1100 | N.S. | 10 | 4900 | 1800 | N.S. |
| Platelet count/mm³ | 10 | 178000 | 26000 | 19 | 171000 | 68000 | N.S. | 10 | 227000 | 47000 | N.S. |
| 4th Course | | | | | | | | | | | |
| Haemoglobin g/100 ml | 8 | 10·9 | 0·9 | 17 | 11·4 | 1·1 | N.S. | 8 | 11·8 | 0·8 | N.S. |
| Total white count/mm³ | 8 | 4300 | 1300 | 17 | 4400 | 1100 | N.S. | 8 | 5200 | 1400 | N.S. |
| Platelet count/mm³ | 8 | 171000 | 43000 | 17 | 162000 | 48000 | N.S. | 8 | 194000 | 48000 | N.S. |

Key as in Table II.

comparable protective effect on the target tissue. Any effect from the anabolic agents would therefore have to be additional to the sparing action of the folinic acid.

Nandrolone decanoate and oxymetholone are both synthetic steroids with a high anabolic and low androgenic activity. Nandrolone decanoate is also a long acting preparation. Both have been shown to have an effect on erythropoiesis (Turner, 1966; Sanchez-Medal *et al.*, 1964).

*Haemoglobin.*—Although the group of patients studied here allowed us to use a standardized therapeutic regimen and the results appear to be significant for the first 2 treatment courses, many other factors make it difficult to place any importance on these results. These factors include: (1) In other studies using anabolic steroids in the treatment of refractory anaemias, no effect on erythropoiesis has been noted until 8 weeks after commencement of therapy (Kennedy, 1962;; Sanchez-Medal *et al.*, 1969); (2) higher doses of anabolic steroids have been used in these studies (Turner, 1966; Sanchez-Medal *et al.*, 1969); (3) malignant disease itself may be accompanied by an anaemia; (4) changes in plasma volume; (5) variable amounts of bleeding from trophoblastic tumours; (6) unequal distribution of pre-treatment haemoglobin values in the present study; (7) transfusions given to several patients during the first 7 days of therapy (no patient received other haematinic therapy); (8) variable nutritional status; (9) variable factors associated with trophoblastic disease and known to have an effect on erythropoiesis, such as oestrogen and thyroxine levels (Bagshawe, 1969; Wintrobe, 1967).

The haemoglobin trend in patients in all groups was upwards towards normal values, and this was so even in those with an initial low haemoglobin level without transfusion or haematinic therapy and

TABLE V.—*To Compare the Time Interval Between the Nadir Total White Count and the Return to Pre-treatment Count in Association With the First Course of Therapy*

| | Control | Nandrolone decanoate | Oxymetholone |
|---|---|---|---|
| (a) Number of patients | 10 | 19 | 12 |
| (b) Mean nadir after commencing therapy (days) | 10 | 10 | 9 |
| (c) Mean return to normal (days) | 18 | 15 | 14·5 |
| (d) Mean time interval (b→c) (days) | 8±2·5 (S.D.) | 5±2·3 ($P = <0·005$) | 5·5±2·5 ($P = <0·025$) |

TABLE VI.—*To Compare the Time Interval Between the Nadir Platelet Count and the Return to Pre-treatment Count in Association With the First Course of Therapy*

| | Control | Nandrolone decanoate | Oxymetholone |
|---|---|---|---|
| (a) Number of patients | 10 | 19 | 12 |
| (b) Mean nadir after commencing therapy (days) | 12 | 12·5 | 12 |
| (c) Mean return to normal (days) | 20 | 21 | 20 |
| (d) Mean time interval (b→c) (days) | 8±1·1 (S.D.) | 8·5±1·3 ($P$=N.S.) | 8±1·6 ($P$=N.S.) |

despite cytotoxic therapy. This may be explained by replacement of red cell mass after previous haemorrhage, by improved nutritional status and by successful treatment of the tumour.

It is probably not unreasonable, despite the above, to conclude that the cytotoxic therapy was at least a contributing factor in the initial fall in haemoglobin values observed in some patients; whether the anabolic steroids played any part in its correction is uncertain.

*Leucocyte count.*—Although no detailed analysis of differential leucocyte counts was made in this study, the overall impression was that the fall in total leucocyte count was due to a fall in neutrophil count, the lymphocyte count remaining relatively constant. This has previously been demonstrated (Shaw, 1971). The beneficial effect of nandrolone phenylpropionate on the lymphocyte count in rats treated with chlorambucil has been demonstrated by Johnston and Burn (1966).

The evidence of a normal neutrophil cycle suggests the presence of factors controlling leucocyte production and the rate of their release into the circulation (Morley, 1966). These have been supported by Gordon (1960) in animal experiments. Small doses of cytotoxic drugs exaggerate this naturally occurring neutrophil cycle and cell counts taken at random may thus be taken either at the peak or trough of a cycle. Increasing the dosage and duration of cytotoxic drugs causes a marked decrease in production of neutrophils and a sustained non-cyclical neutropenia (Morley and Stohlman, 1970; King-Smith and Morley, 1970). During the first course of cyclical methotrexate–folinic acid therapy, a nadir leucocyte count was reached on Day 10 of treatment and this was followed by a rise to pre-treatment values with no overshoot. A similar phenomenon occurs in association with the second course of therapy although the nadir reached is somewhat higher. Following this, the leucocyte count tends to stabilize at a below-normal level (Shaw, 1971). The beneficial effect of anabolic steroids might then be either to minimize the initial leucocyte nadir or to cause stabilization of the leucocyte count at a higher value. Neither of these can be confirmed in the present study although it is possible that a longer study period might yield different results.

The interval between nadir and return to pre-treatment values in those patients receiving the anabolic steroids was significantly shorter than in the controls. This has previously been reported by Horn, Robinson and Hochman (1968) in a study of patients receiving various cytotoxic agents, plus or minus radiotherapy, together with testosterone. The clinical importance of this finding remains to be evaluated.

*Platelet count.*—In the present study, no beneficial effect of anabolic steroids on the platelet count could be shown. The previously reported initial thrombocytopenia followed by rebound thrombocytosis is confirmed (Berlin *et al.*, 1963).

In conclusion, the present study does not lend much support for the use of anabolic steroids in an attempt to minimize marrow toxicity during cytotoxic chemotherapy. Nevertheless, it is a limited short-term study on a selected group of patients being treated with cyclical methotrexate–folinic acid. Further studies will be necessary before a conclusion can be reached, but the difficulties inherent in finding standardized groups of patients with malignant disease for long-term study are numerous. Also, account has to be taken of the report by Delamore and Geary (1971) that 4 patients with aplastic anaemia developed acute myeloblastic leukaemia after treatment with oxymetholone.

We wish to thank Dr G. D. Pegrum and Dr Marion S. Edwards for their kind co-operation in providing the haematological data. We also thank Dr R. D. Slack of Organon Laboratories for supplying Decadurabolin and for a grant in support of technical assistance.

### REFERENCES

BAGSHAWE, K. D. (1969) *Choriocarcinoma.* London: Edward Arnold.

BERLIN, N. I., RALL, D., MEAD, J. A. R., Freireich E. J., SCOTT, E-VAN, HERTZ, R. & LIPSETT, M. B. (1963) Folic Acid Antagonists—Effects on the Cell and the Patient. *Ann. intern. Med.*, **59**, 931.

DACIE, J. V. & LEWIS, S. M. (1968) *Practical Haematology.* 4th Ed. London: Churchill.

DELAMORE, I. W. & GEARY, C. G. (1971) Aplastic Anaemia, Acute Myeloblastic Leukaemia and Oxymetholone. *Br. med. J.*, ii, **743**.

GARBY, L. (1970) The Normal Haemoglobin Level. *Br. J. Haemat.*, **19**, 429.

GARDNER, F. H. & PRINGLE, J. C. (1961) Androgens and Erythropoiesis. *Archs intern. Med.*, **107**, 846.

GORDON, A. S. (1960) Humoral Influences on Blood Cell Formation and Release. Ciba Foundation Symposium on Haemopoiesis.

HORN, Y., ROBINSON, E. & HOCHMAN, A. (1968) Leukopoietic Stimulation by Testosterone in Cancer Patients Treated by Chemotherapy and Irradiation. *Radiol. clin. biol.*, **37**, 29.

JOHNSTON, I. D. A. & BURN, J. I. (1966) The Effect of Nandrolone on the White Cell Counts of Rats Treated with Chlorambucil. Organon Sheffield Symposium: The Value of Cytotoxic Agents and Anabolic Steroids in the Treatment of Advanced Malignant Disease.

KENNEDY, B. J. (1962) Stimulation of Erythropoiesis by Androgenic Hormones. *Ann. intern. Med.*, **57**, 917.

KING-SMITH, E. A. & MORLEY, A. A. (1970) Computer Simulation of Granulopoiesis. Normal and Impaired Granulopoiesis. *Blood*, **36**, 254.

MORLEY, A. A. (1966) A Neutrophil Cycle in Healthy Individuals. *Lancet*, ii, 1220.

MORLEY, A. A. & STOHLMAN, F. (1970) Cyclophosphamide Induced Cyclical Neutropenia. *New Eng. J. Med.*, **282**, 643.

SANCHEZ-MEDAL, L., PIZZUTO, J., TORRE-LOPEZ, E. & DERBEZ, R. (1964) Effect of Oxymetholone in Refractory Anaemia. *Archs. intern. Med.*, **113**, 721.

SANCHEZ-MEDAL, L., GOMEZ-LEAL, A., DUARTE, LORENZO & RICO, MARIA GUADALUPE (1969) Anabolic Androgenic Steroids in the Treatment of Acquired Aplastic Anaemia. *Blood*, **34**, 283.

SHAW, M. T. (1971) Bone Marrow Function During and Following Cytotoxic Chemotherapy in Patients with Trophoblastic Tumours. M.D. Thesis, Newcastle University.

TURNER, R. L. (1966) Combined Androgenic and Cytotoxic Therapy in Carcinoma of Breast. Organon Sheffield Symposium: The Value of Cytotoxic Agents and Anabolic Steroids in the Treatment of Advanced Malignant Disease.

WHYTE-WATSON, G. & TURNER, R. L. (1959) Breast Cancer—a New Approach to Therapy. *Br. med. J.*, i, 1315.

WILLIAMSON, C. S. (1916) Influence of Age and Sex on Haemoglobin. *Archs intern. Med.*, **18**, 505.

WINTROBE, M. M. (1967) *Clinical Haematology.* London: Kimpton.